# Intensive Treatment of Organic Wastewater by Three-Dimensional Electrode System within Mn-Loaded Steel Slag as Catalytic Particle Electrodes

**DOI:** 10.3390/molecules29050952

**Published:** 2024-02-21

**Authors:** Xu Ren, Haifeng Fu, Danni Peng, Meng Shen, Peixin Tang, Kai Song, Bo Lai, Zhicheng Pan

**Affiliations:** 1 School of Architecture and Civil Engineering, Chengdu University, No. 2025, Chengluo Road, Chengdu 610106, China; renxu@cdu.edu.cn (X.R.);; 2 Postdoctoral Research Station in Environmental Science and Engineering, Sichuan University, No. 24, South Section of First Ring Road, Chengdu 610065, China; laibo@scu.edu.cn; 3 Postdoctoral Research Station of Haitian Water Group Co., Ltd., AVIC International Exchange Center, North Section of Yizhou Avenue, Chengdu 610041, China; 202121000322@stu.swpu.edu.cn; 4 School of Energy and Power Engineering, Xihua University, No. 9999, Hongguang Avenue, Chengdu 610039, China

**Keywords:** three-dimensional electrochemical reactor (3DER), transition metals, advanced oxidation process (AOPs), refractory organics, removal efficiency

## Abstract

Developing a green, low-carbon, and circular economic system is the key to achieving carbon neutrality. This study investigated the organics removal efficiency in a three-dimensional electrode reactor (3DER) constructed from repurposed industrial solid waste, i.e., Mn-loaded steel slag, as the catalytic particle electrodes (CPE). The CPE, a micron-grade material consisting primarily of transition metals, including Fe and Mn, exhibited excellent electric conductivity, catalytic ability, and recyclability. High rhodamine B (RhB) removal efficiency in the 3DER was observed through a physical modelling experiment. The optimal operating condition was determined through a single-factor experiment in which 5.0 g·L^−1^ CPE and 3 mM peroxymonosulfate (PMS) were added to a 200 mL solution of 10 mM RhB under a current intensity of 0.5 A and a 1.5 to 2.0 cm distance between the 2D electrodes. When the initial pH value of the simulated solution was 3 to 9, the RhB removal rate exceeded 96% after 20 min reaction. In addition, the main reactive oxidation species in the 3DER were determined. The results illustrated that HO• and SO4^•−^ both existed, but that the contribution of SO_4_^•−^ to RhB removal was much lower than that of HO• in the 3DER. In summary, this research provides information on the potential of the 3DER for removing refractory organics from water.

## 1. Introduction

China is a major producer of steel. Large quantities of steel slag (SS) are produced from iron- and steel-making. SS contains a certain amount of valuable elements [1], such as transition metals including Fe, Cr, Ti, and V, and it has therefore been regarded as a reusable resource [2]. However, the reutilization rate of SS is extremely low. Statistics show that the annual production of SS in China was about 153 million tons, and the stockpile was about 1446 million tons in 2022 [3]. Proper treatment of SS is not only essential for environmental protection, but it also may bring economic benefits.

The three-dimensional electrode reactor (3DER) is considered as a frontier advanced wastewater treatment system, with its higher current efficiency and lower energy consumption than conventional electrochemical reactors [4]. Particle electrodes (PE), also known as the third electrode and located between the anode and cathode, can shorten the mass transfer distance and increase the chemical reaction area in the reactor [5]. In recent years, a new role for PE as the catalyst in a 3DER has been widely considered by investigators, because catalytic PE (CPE) can couple the electrochemical and catalytic oxidation systems (also known as a heterogeneous electrochemical advanced oxidation technologies system) to further improve the working efficiency of the 3DER. Therefore, excellent PEs should have both good electrical conductivity and catalytic properties [5,6].

SS can be an ideal raw material for PE, depending on its composition. SS contains many metallic elements and therefore has a certain electrical conductivity. Wang et al. and Zhang et al. proved the feasibility of SS as a source of conductive particles in a 3DER [7,8]. Moreover, the transition metals in SS may make it catalytic. Wang et al., Li et al., Hou et al., and Lai et al. evaluated the catalytic ability of SS, prepared some novel catalysts, and used them to activate oxidants, e.g., PS and H_2_O_2_, for removing organics from water [9,10,11,12]. However, these catalysts were primarily nanoscale materials that easily agglomerated in the electric field, resulting in a substantial loss of catalytic performance. Our previous study investigated the performance of micron CPE, which was prepared from vanadium-titanium magnetite (V-Ti-Fe) [13]. We found that the micron PE (i.e., the V-Ti-Fe) was stable and easy to recycle, but the resource value of these natural materials was high, and it was economically inefficient to use them as PE in engineering applications. In addition, due to the limited metals content in SS, its electrical conductivity was not outstanding, resulting in high reactor energy consumption, which would hinder the application of SS in electrochemical systems. Therefore, modification methods to improve the electrical conductivity of SS are also worthy of investigation. Considering the composition characteristics of SS, loading the inherent transition metals into SS was a feasible modification method.

Therefore, the blast furnace SS produced during V-Ti-Fe smelting was further investigated in this study. This SS has similar composition to V-Ti-Fe, but its catalytic performance was poorer [14]. We prepared Mn-loaded SS (Mn was the transition metal that was inherently contained in SS and was relatively economical) by ultrasonic impregnation and air calcination to improve its electrical conductivity and catalytic properties and used it as a CPE to equip the 3DER. Peroxymonosulfate (PMS) was used as the oxidant and electrolyte in the electrochemical system. The effect of different conditions on simulated contaminant (rhodamine B, or RhB) removal in the 3DER was investigated through a single-factor experiment. In addition, the main reactive oxygen species (ROS) in the 3DER were examined to illustrate how the 3DER works. The findings are expected to provide a feasible method for reusing SS and to lead to a new wastewater treatment strategy based on “treating waste with waste”.

## 2. Results and Discussion

### 2.1. Characteristics of CPE

**Figure 1:**
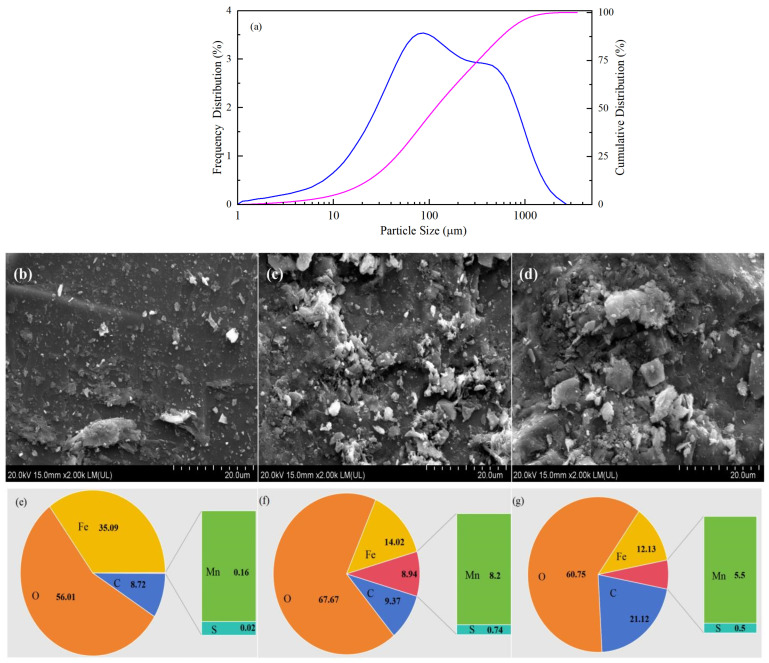
Particle size distribution of CPE (**a**); SEM images of SS (**b**), raw CPE (**c**), and used CPE (**d**); EDS analysis of SS (**e**), raw CPE (**f**), and used CPE (**g**).

The particle size analysis results (Figure 1a) showed that the CPE was a kind of micron material, with the particle size mainly distributed between 40 and 200 μm. The Brunauer–Emmett–Teller (BET) and Langmuir specific surface areas of raw CPE were 0.4014 m^2^ g^−1^ and 0.7043 m^2^ g^−1^, respectively. SEM images showed that the surfaces of the raw CPE (Figure 1c) and the used CPE (Figure 1e) were much rougher than that of the SS sample (Figure 1b), indicating that the modification changed the physical structure of the SS surface. Rough surfaces made it easier for contaminants to adhere, which could facilitate their removal.

The main elements, O, Fe, C, Mn and S, were found through EDS analysis (Figure 1e–g). The metal present in the highest proportion was Fe, which accounted for 14.02% of the CPE. The amount of Mn in CPE was 8.2%, which was much higher than in the SS (0.16%), suggesting that Mn was present at a relatively high level in CPE. The amounts of Fe and Mn in the used CPE decreased slightly to 12.13% and 5.5%, respectively, but the roughness of its surface was comparable to raw CPE. In addition, the EDS-mapping analysis (shown in Figure S3) indicated that many micro-particles containing manganese compounds (the purple particles) existed on the surface of raw CPE, indicating that Mn was successfully loaded onto the surface of the SS through ultrasonic impregnation. However, there were slightly fewer purple particles on the used CPE than on raw CPE, indicating that some Mn was lost during the reaction.

According to the results of XRD analysis (Figure S4), SiO_2_, Fe_2_O_3_, and MnO_2_ were the main components of CPE. Combined with the XRF results (Table 1), the metal elements, such as Fe, Si, Al, K, and Mn, were present in raw CPE. The main compounds also included SiO_2_, Fe_2_O_3_, CaO, Al_2_O_3_, MnO_2_, and TiO_2_. A sufficient content of metal compounds gave the CPE good conductivity, at 88.3158 S/m. In addition, the relative concentrations of the compounds and elements in CPE changed slightly after use. MnO and Mn in CPE were decreased after the reaction, which could be attributed to Mn leaching out. However, the electric conductivity of the used CPE was still good (69.5894 S/m). These phenomena indicated that the stability of CPE was tolerable.

In general, CPE has good electrical conductivity and satisfactory stability. The main active metals (Fe and Mn) and other transition metals (such as Al, Mg, Ti, and V) in CPE all have the catalytic ability for PMS activation without extra energy. Therefore, it is feasible to achieve relatively excellent organics removal efficiency in a 3DER by coupling the activation ability of CPE and the current to PMS.

### 2.2. RhB Removal Efficiencies in the 3DER

#### 2.2.1. Effect of CPE Amount

**Figure 2:**
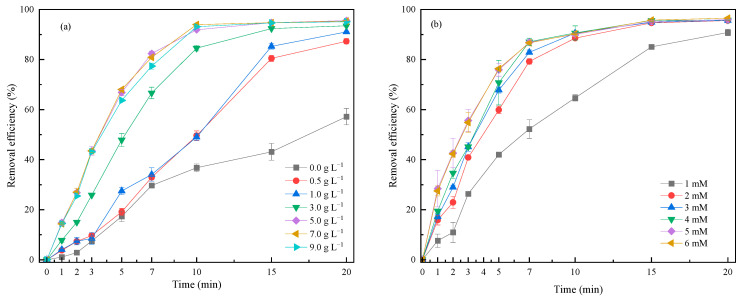

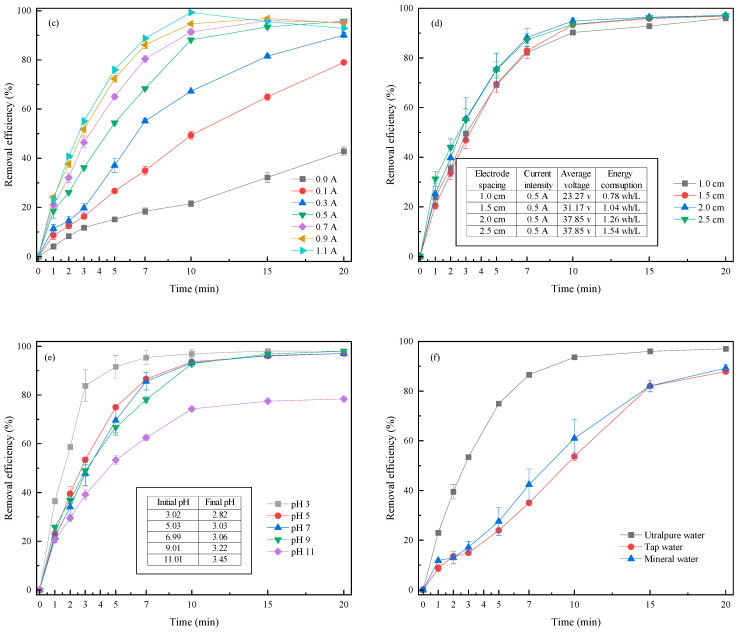
Effects of (**a**) CPE amount; (**b**) PMS concentration; (**c**) current intensity; (**d**) electrode spacing; (**e**) initial pH; and (**f**) real water on RhB removal efficiency in the 3DER.

As we know, the CPE served as both the catalyst and the third electrode in the reaction system, and therefore, its amount was crucial to the treatment efficiency of the 3DER [15,16,17]. On the one hand, if the amounts of oxidant and catalyst are out of proportion, this will affect catalytic oxidation performance. On the other hand, excessive CPE can easily lead to short circuits in the 3DER due to frequent collisions of these conductive particles. The amount of CPE was varied from 0.5 g L^−1^ to 9.0 g L^−1^, with fixed concentrations of RhB (10 mg L^−1^) and PMS (4 mM) and fixed current intensity (0.5 A) and electrode spacing (15 mm). Figure 2a shows that adding CPE to the electrochemical system greatly improved RhB removal. The RhB removal efficiency of the system without CPE was only 57.19% at 20 min, whereas the removal efficiency of the system with 125 mg (0.5 g L^−1^) CPE increased to 87.31%. The removal efficiency increased significantly with an increasing amount of CPE from 0.5 g L^−1^ to 5.0 g L^−1^; however, when the CPE dosage exceeded 5.0 g L^−1^, RhB removal efficiency decreased slightly. Therefore, the suitable amount of CPE was deemed to be 5.0 g L^−1^, with which the RhB removal efficiency was 95.55% after 20 min of electrochemical reaction.

#### 2.2.2. PMS Concentration

Actually, RhB is difficult to remove through an electrochemical process without adding supplementary electrolytes into a reactor, because its aqueous solution is almost nonconductive [14]. Furthermore, RhB cannot be removed through a catalytic oxidation process without any oxidants. Therefore, PMS is similar to a bridge in that it can couple two systems in the 3DER, or in other words, it acts as the oxidant in a catalytic oxidation system and as the electrolyte in an electrochemical system. The effects of the PMS concentration (1, 2, 3, 4, 5, 6 mM) on RhB removal at a fixed RhB concentration (10 mg L^−1^), CPE dosage (5.0 g L^−1^ mM), current intensity (0.5 A), and electrode spacing (15 mm) as the constant parameters were studied. Figure 2b reveals that once PMS was added to the aqueous solution, the RhB removal efficiency rose rapidly, especially within the first 7 min of reaction time. As the PMS concentration increased from 1 mM to 3 mM, the RhB removal efficiency increased from 90.81% to 95.68%. In addition, the increase in PMS concentration improved the conductivity of the solution, thus reducing the average voltage (from 32.28 V to 11.69 V) required to maintain a constant current in the 3DER, which meant that reactor energy consumption was also reduced. However, as the PMS concentration continued to increase (4 to 6 mM), the expected increase in RhB removal efficiency never occurred. On the one hand, the RhB was degraded through a direct oxidation process on the surface of the anode and the anodic surface of the CPE (Equation (1)) [18,19]. On the other hand, PMS was activated by the current and the transition metals in the CPE, generating ROS (e.g., OH and SO_4_^•−^) to oxidize RhB (Equations (2)–(5)) [19]. Nevertheless, the excess PMS could scavenge ROS with its relative high oxidation potential (Equation (6)) [20,21,22]. Meanwhile, excessive SO_4_^•−^ was self-quenched due to its fast generation rate (Equation (7)) [8,23,24,25]. In conclusion, the optimal PMS concentration was 3 mM.

(1) $$\mathrm{RhB}+{129\mathrm{OH}}^{-}\to {28\mathrm{CO}}_{2}+{76H}_{2}O+{2\mathrm{NH}}_{4}^{+}+\frac{1}{2}{\mathrm{Cl}}_{2}+{131e}^{-}$$

(2) $${\mathrm{HSO}}_{5}^{-}+e^{-}\to {\mathrm{SO}}_{4}^{•-}+{\mathrm{OH}}^{-}$$

(3) $${\mathrm{HSO}}_{5}^{-}+e^{-}\to {\mathrm{SO}}_{4}^{2-}{+}^{•}\mathrm{OH}$$

(4) $${\mathrm{HSO}}_{5}^{-}+\equiv M^{n+}\to {\mathrm{SO}}_{4}^{•-}+\equiv M^{(n+1)+}+{\mathrm{OH}}^{-}$$

(5) $$Mn^{(n+1)+}+e^{-}\to Mn^{n+}$$

(6) $${\mathrm{SO}}_{4}^{•-}+{\mathrm{HSO}}_{5}^{-}\to {\mathrm{SO}}_{5}^{•-}+{\mathrm{HSO}}_{4}^{-}$$

(7) $${\mathrm{SO}}_{4}^{•-}+{\mathrm{SO}}_{4}^{•-}\to S_{2}O_{8}^{2-}$$

Notes: ≡ means the surface of CPE; M represents transition metals, e.g., Fe, Mn.

#### 2.2.3. Current Intensity

Current intensity determines the electron transfer and energy consumption of an electrochemical system [26,27]. The current intensities applied were 0, 0.1, 0.3, 0.5 0.7, 0.9, and 1.1 A to research the effect on RhB removal when the RhB concentration was 10 mg L^−1^, the CPE amount was 5.0 g L^−1^, the PMS concentration was 3 mM, and the electrode spacing was 15 mm. Figure 2c shows the influence of the current intensity on RhB removal. The control experiment was carried out under a current intensity of 0.0 A, i.e., the catalytic system was composed of PMS (oxidant) and CPE (catalyst). Here, 42.82% of RhB was removed through 20 min of catalytic oxidation, indirectly illustrating that the CPE had good catalytic ability for PMS activation. The RhB removal efficiency increased significantly after current was applied; for instance, removal improved by nearly 26% when only 0.1 A current was applied in the 3DER. Moreover, RhB removal increased at a higher current intensity due to increased electron transfer. The higher the current intensity, the more ROS could be generated by activating PMS (Equation (5)), and likewise, the more RHB could be decomposed by direct oxidation (Equation (4)). However, energy consumption increased accordingly due to the increase in demand voltage. When the current intensity was 0.1, 0.3, 0.5, 0.7, 0.9, and 1.1 A, the average energy consumption was 0.06, 0.38, 0.94, 1.61, 2.57, and 2.63 Wh L^−1^, respectively, whereas the RhB removal efficiency was 78.97%, 90.01%, 95.72%, 97.26%, 94.25%, and 92.84%. In addition, inflection points of RhB removal efficiency could be observed at current intensities of 0.7, 0.9, and 1.1 A. These occurred because dissolution of the anode was intensified at high current intensities and iron ions were freed from the anode, resulting in increased solution chroma, which affected the RhB removal calculations. These phenomena also showed that a high current was not conducive to stable reactor operation. Therefore, taking these factors into consideration, it was concluded that 0.5 A was an appropriate current intensity for the 3DER.

#### 2.2.4. Electrode Spacing

The electrode spacing was set to 1.0, 1.5, 2.0, and 2.5 cm, with the optimized amount of CPE (5.0 g L^−1^), PMS concentration (3 mM), and current intensity (0.5 A). From Figure 2d, RhB removal efficiencies were 96.01%, 96.88%, 97.12%, and 97.01%, respectively, after 20 min of reaction at different electrode spacings. Although relatively high RhB removal efficiency was achieved at an electrode spacing of 2.0 cm, the effect of electrode spacing on RhB removal was negligible. Usually, close electrode spacing tends to lead to passivation of the anode and the anodic surface of CPE, decreasing the oxidation capacity of the system [28,29]. Although a wider electrode spacing increased energy consumption by increasing the voltage drop at the anode and cathode, the average energy consumption was 0.78, 1.04, 1.26, and 1.54 Wh L^−1^ at different electrode spacings. Therefore, the electrode spacing should be controlled between 1.5 and 2.0 cm to achieve expected RhB removal efficiency while ensuring 3DER stability.

#### 2.2.5. Initial Electrolyte pH

As noted earlier, RhB could be almost completely degraded at the original pH of the 10 mg L^−1^ solution itself (pH of around 6.70) in the 3DER. Both acidic and alkaline conditions may promote PMS activation, according to previous studies. This study investigated the effect of initial pH (3, 5, 7, 9, and 11) on the RhB removal efficiency when the amount of CPE, PMS concentration, current intensity, and electrode spacing were 5.0 g L^−1^, 3 mM, 0.5 A, and 1.5 cm, respectively. The system pH was found to decrease with reaction time; the final pH was 2.82, 3.03, 3.06, 3.22, and 3.45, respectively, which could be attributed to Equation (8). The pH during the electrochemical process remained around 3.0 regardless of the initial pH, which could be related to the 3DER buffering capacity. The RhB removal efficiencies under acidic conditions were higher than those under alkaline conditions, especially within the first 7 min. The best RhB degradation (removal of 97.97% at 20 min) was reached at pH 3.00. Nevertheless, similar removal efficiencies were achieved at the end of the reaction under initial pHs of 3–9 (97.97%, 97.08%, 97.05%, and 96.86%, respectively), illustrating that the 3DRE could be applied over a wide range of initial pHs. Furthermore, a notable decrease in RhB degradation was observed, with only 78.38% RhB removal efficiency, when the initial pH was 11, and some floccus could be observed during the reaction. These phenomena could be attributed to the following reason: (i) the Fe in CPE formed Fe(OH)*_n_* (*n* = 2, 3) colloids [15,29], decreasing the electric conductivity, but releasing H^+^ for neutralization under alkaline conditions [25]. In practice, it is necessary to consider not only treatment efficiency, but also the cost of the drug and the ease of operation. Therefore, it can be recommended that wastewater with a pH value of 3 to 9 needs no adjustment to its initial pH.

(8) $${\mathrm{SO}}_{4}^{•-}+H_{2}O\to {\mathrm{SO}}_{4}^{2-}{+}^{•}\mathrm{OH}+H^{+}$$

#### 2.2.6. Effect of Real Water on 3DER Efficiency

A total of 10 mg L^−1^ of RhB solution was prepared using tap water and mineral water, respectively, after which RhB removal efficiency was monitored under the optimal operating conditions described above to evaluate the performance of the 3DER on pure water. In other words, the initial pH was not adjusted, and the amount of CPE, PMS concentration, current intensity, and electrode spacing were 5.0 g L^−1^, 3 mM, 0.5 A, and 1.5 cm, respectively. Figure 2f shows that the RhB removal efficiency decreased throughout the treatment, especially within the first 10 min. This phenomenon illustrated that the complex components in water had a negative effect on 3DER treatment efficiency. Some inorganic anions (such as NO_3_^−^, CO_3_^2−^, and PO_4_^3−^) and natural organic components (for instance, humic and fulvic acids) in water can react with the main ROS in the 3DER to reduce the oxidative potential, thus inhibiting RhB degradation [26,27]. Fortunately, the 3DER obtained a reasonable RhB removal efficiency (about 85%) in real water after 20 min reaction. This experiment also provided us with an idea for the next step in the research, that is, how to improve 3DER efficiency in practical application.

In addition, we constructed the 3DER using a non-airtight perspex jar in this study, making it difficult to quantify CO_2_ precisely. However, we could still theoretically calculate the amount of CO_2_ produced, because CO_2_ was derived from the complete oxidation of RhB. Therefore, the RhB in 200 mL of RhB solution (10 mg L^−1^) could be completely oxidized to produce 5.14 mg of CO_2_. It can be confirmed that CO_2_ was inevitably produced in the process of treating organic wastewater with 3DER. Therefore, the collection and proper disposal of CO_2_ was our next research topic.

### 2.3. Analysis of the Main Reactive Oxidation Species

**Figure 3:**
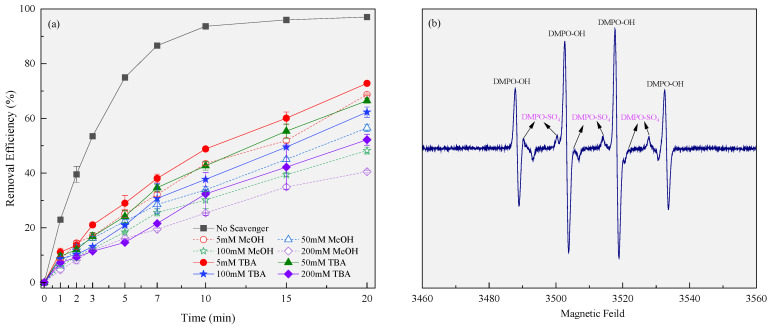
Main ROS in the 3DER determined by adding scavengers (**a**) and EPR detection (**b**).

According to previous research cases, PMS can be activated through electric current or transition metal compounds to form reactive oxidation species (ROS, mainly SO_4_^•−^ and HO•) in the liquid phase [30,31,32]. This study demonstrated that PMS could be activated by the synergistic action of the current and CPE in the 3EDR. First, certain concentrations of radical quenchers, including MeOH (applied as a scavenger for both SO_4_^•−^ and HO•) and TBA (applied as a scavenger for HO•) [33], were added to the 3DER to evaluate their effect on RhB removal, thereby indirectly determining the free radical species in the system. In this experiment, the amounts of scavenger were varied from 5 mM to 50 mM, 100 mM, and 200 mM, with the fixed parameters being the initial pH (adjusted), amount of CPE (5.0 g L^−1^), PMS concentration (3 mM), current intensity (0.5 A), and electrode spacing (1.5 cm). Figure 3a shows that the RhB removal efficiency decreased at higher radical scavenger concentrations. When the same concentrations of MeOH and TBA were added to the 3DER, the RhB removal efficiency of the former was lower, indicating that HO• and SO_4_^•−^ might be produced during the reaction. This trend can be attributed to the fact that the higher the concentrations of the quenchers, the stronger their quench ability will be for radicals. Nevertheless, there were no significant differences in RhB removal efficiency between the two radical scavengers at the same dosage, and therefore, we inferred that the contribution of SO_4_^•−^ to RhB removal was much less than that of HO• in the 3DER. Most of the original SO_4_^•−^ radicals generated in the 3DER were converted into HO• and co-removed RhB. Therefore, EPR was subsequently used to identify ROS directly (Figure 3b). The results showed that both the characteristic peaks of DMPO-OH and DMPO-SO_4_ were present, corroborating our theory.

### 2.4. Recyclability of CPE

**Figure 4:**
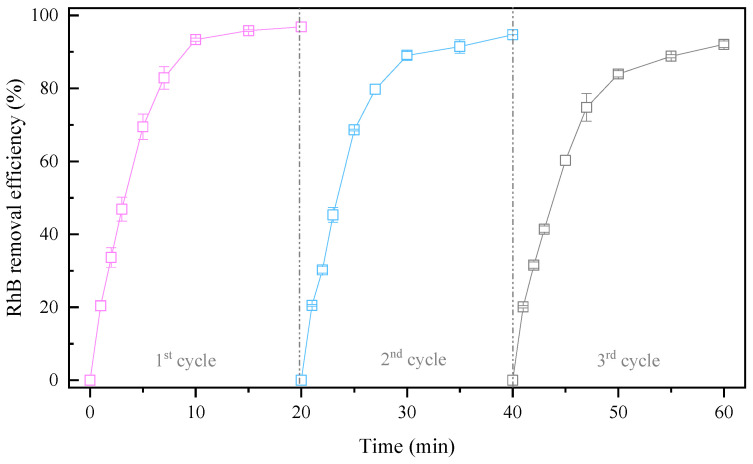
Recyclability of CPE.

CPE was found to have tolerable stability according to an analysis of its components before and after use. To further examine the recyclability of CPE, life tests were performed for three cycles (Figure 4). The CPE was directly separated by magnetic separation after each run, ultrasonically cleaned (power: 200 W, frequency: 40 kHz) to remove adsorbates from the CPE surface, and dried at 105 °C. The RhB removal efficiencies after each cycle were 96.88%, 94.73%, and 92.09%, respectively. Although RhB removal by the 3DER decreased slightly with the number of times of CPE usage, more than 90% of the RhB was still removed. Interestingly, compared to our previous studies (using H_2_O_2_ as the oxidant), although HO• was the major contributor to RHB removal in both systems, markedly fewer flocs were produced during this experiment, and the CPE recovery rate was higher after each cycle. Moreover, 3DER performance in this study was much more stable than in previous studies. This may be attributed to the relatively high utilization rate of HO• for RhB removal in this research case, rather than reaction with Fe in the CPE, illustrating that the 3DER using PMS as the oxidant had better stability and was more suitable for practical application in wastewater treatment.

## 3. Materials and Methods

### 3.1. Samples and Reagents

All SS samples were collected from the smelting process of the same type of steel. We used the quartering method to sample SS from the blast furnace of a steel mill in southwestern China. It was stored in an airtight container after coarse breaking. The chemicals were analytically pure grade or guaranteed reagent grade, as listed in Text S1.

### 3.2. Experimental Procedure

#### 3.2.1. Preparation of Catalytic Particle Electrodes

Each SS sample was broken further with a crusher and sieved through a 100-mesh screen. Then, 300 g of sieved sample were placed with 700 mL deionized water into a 1000 mL beaker for oscillatory cleaning five times, 15 min each time. Subsequently, the sample was dried at 35 °C in an electrically heated air-blowing drier. Next, the dried SS was mixed with 0.5 M of MnSO_4_ solution, stirred, and ultrasonically oscillated for 40 min. The excess liquid was removed, after which the remaining solids were calcined in a muffle furnace at 750 °C for 100 min. After cooling to room temperature, the solids, i.e., the CPE, were taken out and ground with a mortar until the particles were uniform. The CPE was then placed in sealed storage for constructing the 3DER.

#### 3.2.2. Removal Efficiency of 3DER on Organics

A fluid-bed 3DER was constructed using the CPE described above coupled with a mesh stainless-steel anode and cathode of dimensions 35 mm × 25 mm × 1 mm [13,14], as shown in Figure S1. The reactor model was the same as in our previous study. The effects of key factors, including the dosage of CPE (from 0.5 to 9 g L^−1^), PMS concentration (from 1 to 10 mM), current intensity (from 0.1 to 1.1 A), electrode spacing (10 mm, 15 mm, 20 mm, and 25 mm), initial pH of the electrolytes (3, 5, 7, 9, 11), and reaction time (from 0 to 20 min) on RhB removal were investigated through a single-factor experiment, and the optimized reaction condition was determined.

Moreover, the possible mechanism of organics removal in the 3DER was investigated. On the one hand, we added certain concentrations (0, 5, 10, and 50 mM) of free-radical scavenger, including methyl alcohol (MeOH) and tertbutyl alcohol (TBA), to the reaction system and analyzed the effect on RhB removal (initial concentration: 10 mg L^−1^) to indirectly identify the possible existence of ROS. On the other hand, the ROS species during the reaction were directly identified using an electron paramagnetic resonance detector (EPR). In addition, electrolyte samples at reaction times of 1, 2, 3, 5, 7, 10, 15, and 20 min were collected and mixed to analyze the intermediates using a liquid chromatography mass spectrometer (LCMS). All tests were duplicated, and the standard deviations were reported to minimize experimental errors.

### 3.3. Analysis Methods

#### 3.3.1. Characterization of the CPE

Scanning electron microscopy (SEM, GeminisemSEM 300, Zeiss, Ltd., Oberkoche, Germany) and a laser particle size analyzer (Mastersizer 3000, Malvern, UK) were used to analyze the microstructure and particle size distribution of CPE. The elemental composition was investigated by an X-ray spectroscopic analyzer. Micromeritics ASAP 2460 (Micromeritics, Norcross, GA, USA) analyzed the nitrogen gas uptake isotherms to calculate the specific surface area based on the Brunauer–Emmett–Teller (BET) model. The mineral constituents of CPE by X-ray diffraction (XRD, XD-2, Pu Xi, Ltd., Beijing, China) were determined on the CPE, with working conditions of 2 theta (2θ) diffraction within 10°–70° at 0.02° intervals and a scanning speed of 4° min^−1^. In addition, X-ray fluorescence analysis (XRF, Axios, PANalytical, Ltd., Almelo, The Netherlands) was used to determine the compounds and the elemental substances in CPE before and after use.

The electrical resistivity (ρ) of CPE was analyzed using a semiconductor powder resistivity tester (ST-2722, Suzhou Jingge, Ltd., Suzhou, China). Its electric conductivity (K) was calculated using Equation (9):

K = 1/ρ.(9)

#### 3.3.2. RhB Removal Efficiency

RhB is a synthetic dye with a bright pink color and a strong absorption peak at 554 nm wavelength; the absorption value is positively correlated with its concentration. A certain RhB concentration can be determined through the standard calibration curve (Figure S2). The RhB removal efficiency can be evaluated under different reaction conditions through Equation (10):

RhB removal efficiency = (1 − C_t_/C_0_) × 100%, (10)

where C_t_ represents the RhB concentration at a certain reaction time, and C_0_ represents its initial concentration (10 mg L^−1^).

The energy consumption of the 3DER (E, Wh L^−1^) was evaluated through Equation (11), where $\bar{U}$(V) refers to the average voltage throughout the electrochemical reaction, $\bar{U}=\frac{{(U}_{0}+U_{t})}{2}$; I represents current intensity (A); T represents reaction time (h); and V refers to the volume of the solution (L):

(11) $$E=\frac{\bar{U}\mathrm{IT}}{V}$$

#### 3.3.3. Main Reactive Oxidation Species in the 3DER

The effect of different concentrations of free radical scavengers on the reaction was demonstrated by the RhB removal efficiency (Equation (1)). The electron paramagnetic resonance (EPR, Bruker EMXPlus-10/12, Bruker, Germany) was used to characterize the possible free radicals. 5, 5-dimethyl-1-pyrrolidine N-oxide (DMPO) was used in the EPR measurement as the spin-trapping agent. The standard EPR spin-trapping mixture contained 100 mM DMPO, 5.0 g L^−1^ CPE, and 3 mM PMS in ultrapure water, with an electrical current intensity of 0.7 A and a 15 mm electrode spacing.

## 4. Conclusions

The major findings in this study can be summarized as follows:

(1) Mn-loaded SS was a micron-level material with satisfactory electric conductivity, good catalytic ability, and reusability, consisting primarily of O, Fe, C, Mn, and S. It can be used as the CPE for a 3DES fitted with mesh stainless-steel 2D electrodes.
(2) The optimal operating condition was determined through a single-factor experiment, adding 5.0 g·L^−1^ CPE and 3 mM PMS into 200 mL of a 10 mM RhB solution under a current intensity of 0.5 A and a 1.5 to 2.0 cm distance between the 2D electrodes. When the initial pH of the simulated solution was 3 to 9, the RhB removal rate reached more than 96% after 20 min reaction. However, the RhB removal efficiency was decreased in pure water (to about 85%) due to the quenching reaction of some components with ROS in water.
(3) HO• and SO_4_^•−^ were the main ROS in the 3DER, although the contribution of SO_4_^•−^ to RhB removal was much lower than that of HO• during the reaction.

## Figures and Tables

**Table 1:** Compounds and elementary substances in raw and used CPE.

Compounds	Raw CPE	Used CPE	Elementary Substances	Raw CPE	Used CPE
	Concentration (%)	Concentration (%)		Concentration (%)	Concentration (%)
SiO_2_	23.37	25.75	Fe	19.77	19.77
Fe_2_O_3_	18.91	18.86	Si	12.72	14.06
Al_2_O_3_	6.68	7.21	Ca	4.00	1.80
CaO	4.20	1.88	Al	3.99	4.31
K_2_O	2.87	3.17	K	3.07	3.42
SO_3_	1.87	0.46	Mn	1.55	1.07
MnO	1.37	0.94	S	0.93	0.23
MgO	1.33	1.39	Ti	0.90	0.96
Na_2_O	1.09	1.21	Mg	0.89	0.93
TiO_2_	1.08	1.17	Na	0.88	0.98

## Data Availability

The data presented in this study are available in article and Supplementary Materials.

## Supplementary Materials

The following supporting information can be downloaded at: https://www.mdpi.com/article/10.3390/molecules29050952/s1.
