# Supplementary material for: Intensive Treatment of Organic Wastewater by Three-Dimensional Electrode System within Mn-Loaded Steel Slag as Catalytic Particle Electrodes
Source: Molecules. 2024 Feb 21;29(5):952. doi: 10.3390/molecules29050952 (PMC10934177; doi:10.3390/molecules29050952)

Supplementary Materials for

Intensive Treatment of Organic Wastewater by Three-Dimensional Electrode System within Mn-Loaded Steel Slag as Catalytic Particle Electrodes

1. Supplementary information of text

Text S1

Rhodamine B (RhB, $C_{28}H_{31}ClN_2O_3$), with a molecular of 479.01 and ≥99.0% purity, was purchased from Macklin and used as received. Potassium peroxymonosulfate (PMS) was guarantee reagent grade. Other chemicals, including manganese sulfate ($MnSO_4$), sodium sulfates (Na_2SO_4), sodium thiosulfate ($Na_2S_2O_3$), sodium hydroxide (NaOH), sulfate acid (H_2SO_4), tert-butyl alcohol (TBA) and methyl alcohol (MeOH) were analytically pure grade. All chemicals were supplied by Aladdin.

2. Supplementary information of figures

Figure S1 Schematic of the experimental setup.

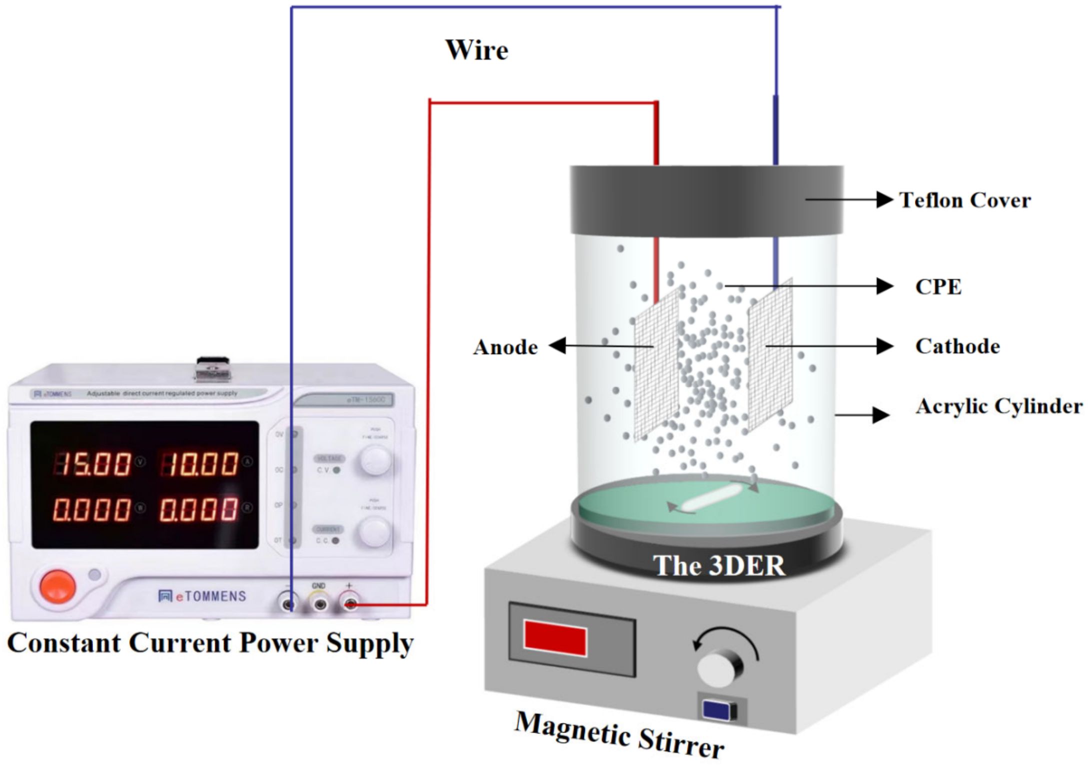

Figure S2. The standard calibration curve of RhB concentration

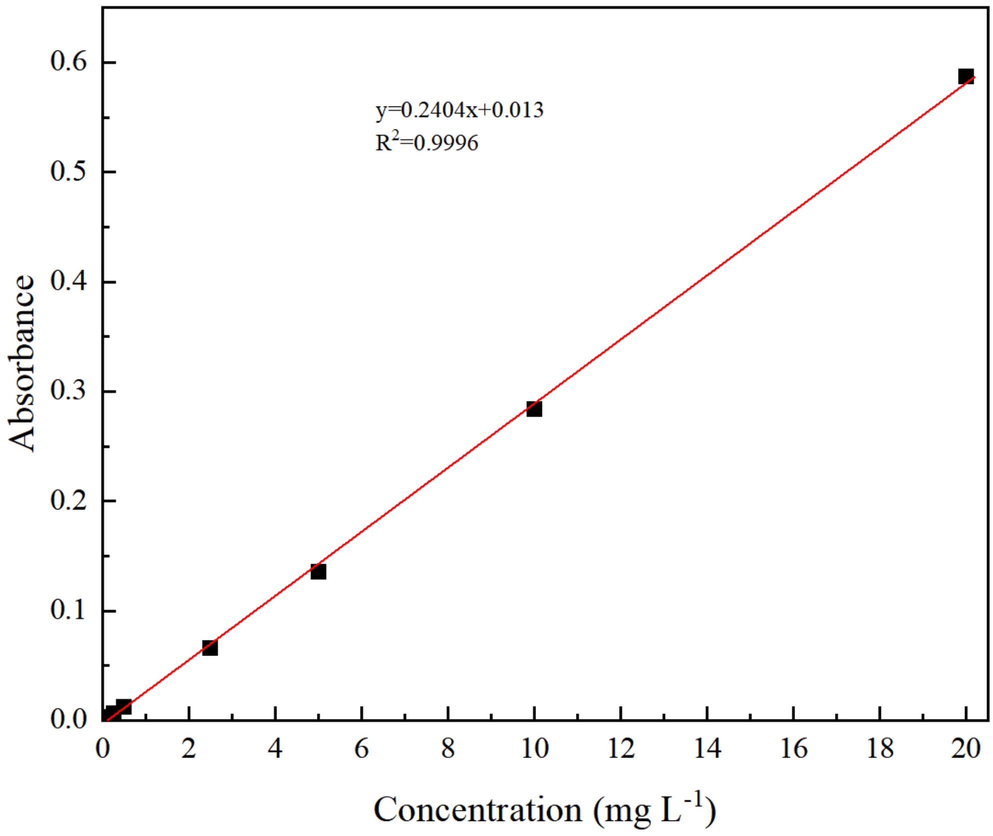

Figure S3. The EDS-mapping results of ss, raw CPE and used CPE

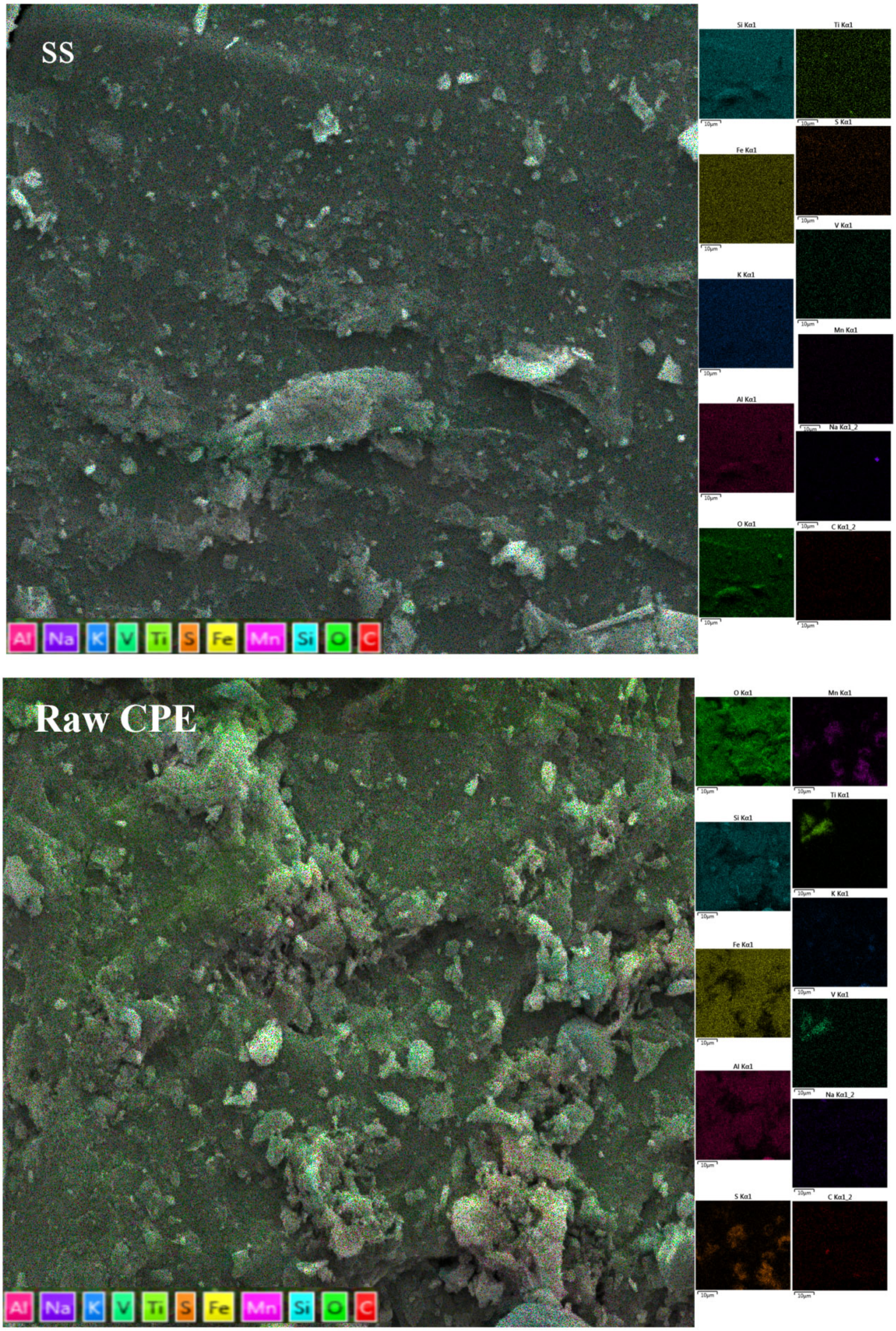

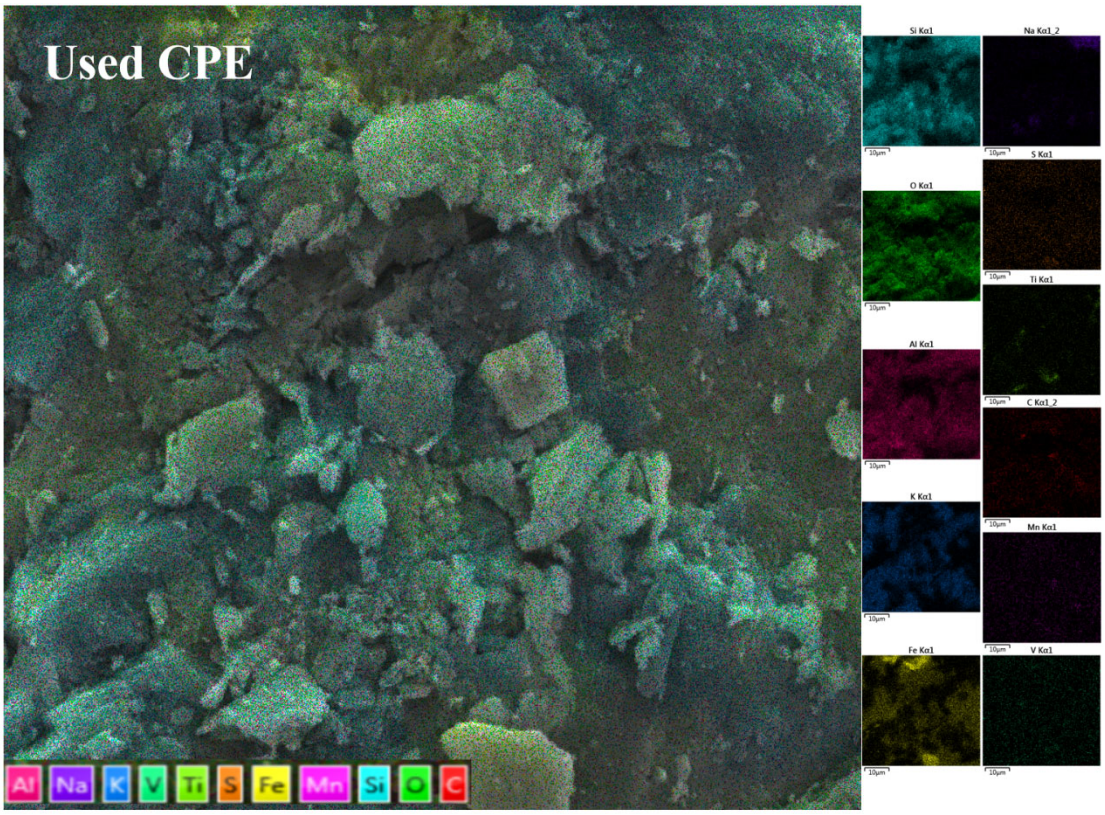

Figure S4. XRD analysis of CPE

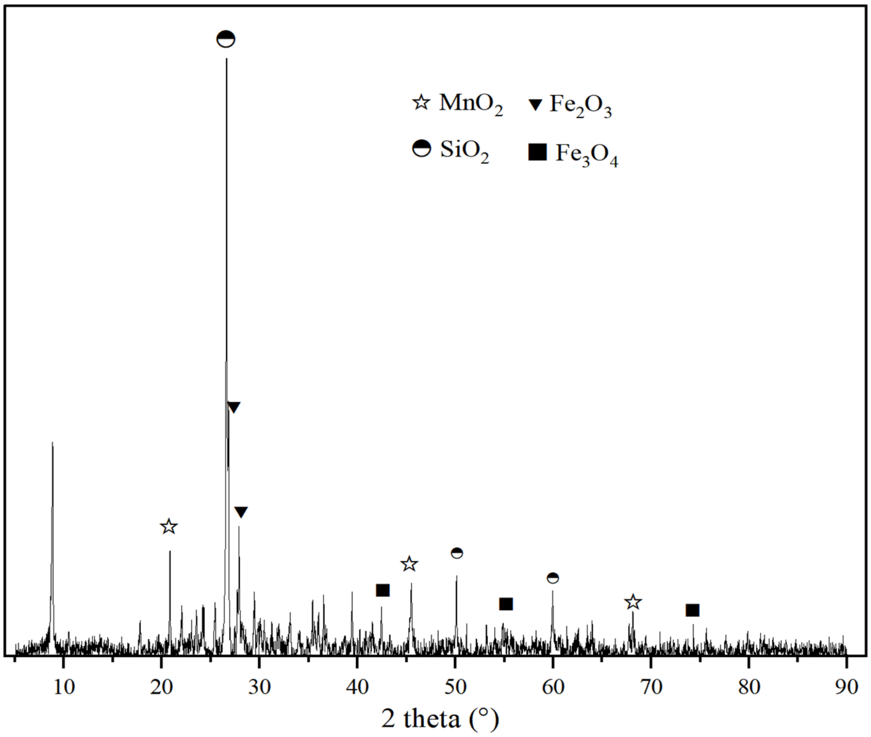

Supplement: Supplementary file 1 [file molecules-29-00952-s001.zip › molecules-2826065-supplementary.pdf]
